# Differences in MHC and TAP-1 expression in cervical cancer lymph node metastases as compared with the primary tumours.

**DOI:** 10.1038/bjc.1994.231

**Published:** 1994-06

**Authors:** F. V. Cromme, P. F. van Bommel, J. M. Walboomers, M. P. Gallee, P. L. Stern, P. Kenemans, T. J. Helmerhorst, M. J. Stukart, C. J. Meijer

**Affiliations:** Institute for Pathology, Free University Hospital, Amsterdam, The Netherlands.

## Abstract

**Images:**


Br. J. Cancer (1994), 69, 1176–1181 © Macmillan Press Ltd., 1994

### Differences in MHC and TAP-1 expression in cervical cancer lymph node metastases as compared with the primary tumours

F.V. Cromme[1], P.F.J. van Bommel[2], J.M.M. Walboomers[1], M.P.W. Gallee[3], P.L. Stern[4], P. Kenemans[5], Th.J.M. Helmerhorst[5], M.J. Stukart[1] & C.J.L.M. Meijer[1]

[1]*Institute for Pathology, Section of Molecular Pathology, Free University Hospital, De Boelelaan 1117, 1081 HV Amsterdam, The Netherlands;* [2]*Department of Gynaecology, Ignatius Hospital, Molengracht 21, 4818 CK Breda, The Netherlands;* [3]*Department of Pathology, Anthoni van Leeuwenhoek Hospital, Plesmanlaan 121, 1066 CX Amsterdam, The Netherlands;* [4]*Department of Immunology, Paterson Institute for Cancer Research, Christie Hospital (NHS) Trust, Wilmslow Road, Manchester M20 9BX, UK;* [5]*Department of Gynaecology, Free University Hospital, De Boelelaan 1117, 1081 HV Amsterdam, The Netherlands.*

**Summary** In previous studies we have shown down-regulation of class I major histocompatibility complex (MHC) expression in a significant proportion of primary cervical carcinomas, which was found to be strongly correlated with loss of expression of the transporter associated with antigen presentation (TAP). By contrast, class II MHC expression was frequently up-regulated on neoplastic keratinocytes in these malignancies. In order to investigate whether these changes are associated with biological behaviour of the tumours, 20 cervical carcinomas were analysed for MHC (HLA-A, HLA-B/C, HLA-DR) and TAP-1 expression in the primary tumours and in lymph node metastases by immunohistochemistry. The results showed a significant increase in the prevalence of HLA-A and HLA-B/C down-regulation in metastasised neoplastic cells as compared with the primary tumour ($P = 0.01$). In all cases this was accompanied by loss of TAP-1 expression. Up-regulated HLA-DR expression was found exclusively in primary tumours and was absent in the corresponding metastases ($P = 0.002$). These data are consistent with the hypothesis that loss of TAP-1 and the consequent down-regulation of class I MHC expression provides a selective advantage for neoplastic cervical cells during metastasis. Furthermore, the lack of class II MHC expression in metastasised cells either reflects a different local lymphokine production or indicates that these cells may have escaped $CD4^+$ cytotoxic T-lymphocyte (CTL)-mediated killing.

Recognition of antigen by the cellular immune system is dependent on the function of major histocompatibility complex (MHC) cell-surface molecules, encoded by the *HLA* genes in human. Class I MHC molecules preferably present endogenous proteins to $CD8^+$ cytotoxic T lymphocytes (CTLs) as small antigenic peptides (Townsend *et al.*, 1985). These peptides are generated by degradation of proteins in the cytosol, possibly involving the proteasome complex (Goldberg & Rock, 1992). Subsequently peptides are translocated into the endoplasmic reticulum (ER) by the transporter associated with antigen presentation (TAP) (Trowsdale *et al.*, 1990; Spies *et al.*, 1990; Neefjes *et al.*, 1993; Shepherd *et al.*, 1993). The population of class I MHC molecules with allelic variation in the vicinity of the antigen-binding cleft allows a wide variety of peptide sequences to bind to class I MHC in the ER (Falk *et al.*, 1990), resulting in their presentation at the cell surface. The MHC–antigenic peptide complex can then activate specific CTLs to proliferate, and these will eventually destroy the antigen-presenting cell (Townsend *et al.*, 1985).

Down-regulation of class I MHC expression has frequently been reported in human malignancies (Ruiz-Cabello *et al.*, 1991). Such down-regulation would supply a selective growth advantage for malignant tumours by allowing neoplastic cells to escape CTL-mediated destruction. Indeed when class I MHC expression is lost in breast (Concha *et al.*, 1991) or laryngeal (Esteban *et al.*, 1989) carcinomas, a significantly poorer clinical outcome is found.

Expansion of an antigen-specific CTL population *in vivo* mostly depends on interleukin 2 (IL-2) production by activated T-helper (Th) cells. This activation is mediated by recognition of opsonised exogenous or endogenous antigen in the context of class II MHC molecules, expressed on professional antigen-presenting cells (APCs). In addition, class II MHC expression can be up-regulated on non-professional APCs, as has been observed in a variety of human malignancies (Sakai *et al.*, 1987; van den Ingh *et al.*, 1987; Paterson *et al.*, 1988). Such up-regulated class II MHC expression has been reported to have a positive (Esteban *et al.*, 1990) or negative (Ruiter *et al.*, 1986) effect with regard to prognosis.

Correspondence: F.V. Cromme.
Received 10 November 1993; and in revised form 11 February 1994.

Changes in both class I and II MHC expression have been found in a substantial number of premalignant and malignant lesions of the uterine cervix, containing human papillomavirus (HPV) genotypes (Connor & Stern, 1990; Glew *et al.*, 1992, 1993; Cromme *et al.*, 1993*a*). Down-regulation of class I MHC expression in cervical carcinomas is apparently post-transcriptionally controlled (Cromme *et al.*, 1993*b*). This is consistent with the observation that class I MHC loss is frequently associated with a failure to detect one of the subunits of the transporter associated with antigen presentation (TAP-1) (Cromme *et al.*, 1993*c*).

However, little is known about the biological relevance of the observed TAP-1 loss and the consequent down-regulation of class I MHC expression in cervical malignancies. Also, the influence of up-regulation of class II MHC is still poorly understood. Evidence of progression is provided by the presence of metastases in the draining lymph nodes. Therefore we investigated in this study whether immmunohistochemically determined changes in MHC and TAP-1 expression are related to metastasis in HPV-positive cervical carcinomas.

The results showed that loss of class I MHC expression was significantly more frequent in metastases as compared with the primary tumour, and was always associated with loss of TAP-1 expression. Furthermore up-regulated HLA-DR expression was shown to be restricted to the primary tumour site, since no metastasised neoplastic cells were found to express HLA-DR.

### Materials and methods

### *Tissues*

Tissues were obtained from patients with cervical carcinoma stage IB or IIA who were treated at the Free University Hospital in Amsterdam. These patients underwent radical surgery combined with pelvic lymph node dissection. Twenty patients diagnosed as having neoplastic tumour cells in the locoregional lymph nodes were entered into the study. From all primary tumours and lymph node metastases representative paraffin tissue blocks were selected and serially cut to 4 μm sections. To ensure the presence of tumour, the first and last slide from each series were stained routinely with haematoxylin-eosin (H&E) and subjected to microscopic examination. Slides of primary tumours and metastatic lymph nodes were used for immunohistochemistry and polymerase chain reaction (PCR) analysis.

### *HPV DNA detection*

The presence of HPV genotypes was analysed by PCR analysis on five tissue sections as described previously (Cromme *et al.*, 1993*a*), employing a general primer (GP5/6), type-specific primer-mediated PCR strategy (Snijders *et al.*, 1990; van den Brule *et al.*, 1990; Walboomers *et al.*, 1992). DNA from SiHa and HeLa cell lines, which contain HPV 16 and 18 DNA respectively, served as positive controls for the HPV PCR. Quality of target DNA for PCR purposes was analysed by PCR using human β-globin gene-specific primers. Liver sections, cut between carcinoma samples, were analysed by PCR to check for contamination during tissue processing and were consistently negative for HPV, while the β-globin primer set gave a positive PCR result.

### *Immunohistochemistry*

Immunohistochemical staining was performed as previously described (Cromme *et al.*, 1993*a*). Briefly, sections adhered to coated [0.1% (w/v) poly-L-lysine) slides were deparaffinised with xylene, rehydrated and endogenous peroxidase was blocked by incubating for 30 min with methanol, containing 0.3% (v/v) hydrogen peroxide. After rinsing in phosphate-buffered saline, pH 7.4 (PBS), sections of each biopsy were processed according to the appropriate protocol for the different primary antibodies (Table I).

After pretreatment and washing repeatedly in PBS, sections were preincubated with normal goat (1:20) or horse (1:50) serum, depending on the secondary antibodies used, for 15 min and then incubated with primary antibodies (Table I).

Bound murine antibodies were detected with a biotinylated horse anti-mouse Ab, 1:500 (Vector Lab., Burlingame, CA, USA), bound rabbit antibodies with a biotinylated goat anti-rabbit Ab, 1:500 (Vector Lab.). Detection of binding of secondary antibody was performed using horseradish peroxidase coupled to avidin-biotin complex, 1:500 (Vector Elite, Vector Lab.), after which the complex was visualised using diaminobenzidine and hydrogen peroxide. Slides were counterstained with haematoxylin, dehydrated and mounted in Depex. The percentage of neoplasteic cells that show staining for class I and II MHC and TAP-1 was determined, with normal epithelium and cells of the immune system serving as positive internal controls for class I MHC and TAP-1 and columnar epithelium and infiltrating immune cells for class II. Primary tumours and lymph node metastases were classified according to the percentage of neoplastic cells that were stained. Three expression patterns were discerned for class I MHC and TAP-1 expression: positive (+), when virtually all neoplastic cells show membranous staining;

**Table I** Features of primary antibodies used in this study

| *Name* | *Type* | *Antigen* | *Source* | *Pretreatment* | *Titre* | *Incubation* |
|---|---|---|---|---|---|---|
| Pankeratin | Polyclonal rabbit | Broad-spectrum cytokeratins | Dakopatts, Glostrup, Denmark | 30 min, 37°C with 0.5% trypsin[a] | 1:400 | RT, 60 min |
| LN3 | MAb mouse | HLA-DR | Biotech, Breieich, Germany | 2 × 5 min, 100°C in lead thiocyanate[b] | 1:25 | 4°C overnight |
| HC-A2 | MAb mouse | HLA-A | Stam *et al.* (1986) | 2 × 5 min, 95°C in TUF[c] | 1:500 | RT, 60 min |
| HC10 | MAb mouse | HLA-B/C | Stam *et al.* (1986) | None | 1:1,000 | RT, 60 min |
| TAP-1 | Polyclonal rabbit | Human TAP-1 | Cromme et al. (1993*c*) | 2 × 5 min, 95°C in TUF | 1:400 | RT, 60 min |

[a]Digestion with 0.3% (w/v) trypsin in 0.5% calcium chloride (pH 7.8). [b]Saturated solution of lead thiocyanate. [c]TUF, target unmasking fluid (Kreatech, Amsterdam, The Netherlands) applied according to manufacturer's instructions. RT, room temperature; MAb, monoclonal antibody.

**Table II** Immunohistochemical staining patterns for class I and II MHC and TAP-1 in primary carcinomas and lymph node metastases (LN)

| | | | *HLA-A* | | *HLA-B/C* | | *TAP-1* | | *HLA-DR* | | *MHC-I* |
|---|---|---|---|---|---|---|---|---|---|---|---|
| *Patient* | *Stage* | *HPV* | *Tumour* | *LN* | *Tumour* | *LN* | *Tumour* | *LN* | *Tumour* | *LN* | *Trend*[a] |
| 77-1856 | IIA | 33 | + | + | + | + | + | + | + | − | = |
| 78-1019 | IB | 18 | + | + | + | + | + | + | + | − | = |
| 70-2049 | IB | X | + | +/− | + | +/− | + | +/− | + | − | ↓ |
| 72-5453 | IIA | 16 | + | +/− | + | +/− | + | +/− | + | − | ↓ |
| 73-4950 | IB | ND | + | +/− | + | +/− | + | +/− | − | − | ↓ |
| 78-3361 | IIA | 16 | + | +/− | + | +/− | + | +/− | − | − | ↓ |
| 70-2774 | IB | ND | +/− | +/−[b] | +/− | +/− | +/− | +/− | + | − | = |
| 71-3857 | IIA | 16 | +/− | − | +/− | − | +/− | − | − | − | ↓ |
| 76-3334 | IIA | 16 | +/− | − | +/− | − | +/− | − | + | − | ↓ |
| 76-3711 | IB | 16 | +/− | − | +/− | − | +/− | − | − | − | ↓ |
| 78-1401 | IB | 31 | +/− | − | +/− | − | +/− | − | − | − | ↓ |
| 83-3968 | IB | 16 | +/− | − | +/− | − | +/− | − | − | − | ↓ |
| 72-831 | IB | 16 | + | −[b] | + | − | + | − | + | − | ↓↓ |
| 76-1849 | IB | 18 | + | − | + | − | + | − | + | − | ↓↓ |
| 79-766 | IB | 16 | + | − | + | − | + | − | − | − | ↓↓ |
| 80-5041 | IB | 18 | +/− | +/− | +/− | +/− | +/− | +/− | − | − | = |
| 70-746 | IIA | 16 | − | − | − | − | − | − | − | − | = |
| 70-2486 | IIA | 16 | − | − | − | − | − | − | − | − | = |
| 75-184 | IIA | 16 | − | − | − | − | − | − | + | − | = |
| 77-6541 | IIA | 16 | − | − | − | − | − | − | − | − | = |

[a]MHC-I trend: =, similar staining pattern for HLA and TAP-1 in primary tumour and lymph node; ↓, more down-regulation in lymph node than in primary tumour (+/− compared with +, or − compared with +/−); ↓↓, negative MHC-I expression in lymph node while primary tumour is positive. [b]Weakly positive cytoplasmic staining. Lesions were scored as + when virtually all neoplastic cells showed positive membranous staining, − when virtually all neoplastic cells showed strongly reduced or negative staining as compared with internal control cells and +/− when positively staining tumour areas were observed adjacent to negative areas. ND, not determinable.

negative (−), when virtually all neoplastic cells show strongly reduced to negative staining; heterogenous (±), when groups of positively staining neoplastic cells were observed adjacent to groups of negative cells, the latter generally accounting for 25–75% of the neoplastic cells in a section. For class II MHC expression, lesions were classified as positive when areas of neoplastic cells, generally comprising 25% or more of the neoplastic cells in a section, showed positive staining for class II MHC. When only a few scattered neoplastic cells were stained, lesions were not scored as up-regulated.

Statistical analysis of the frequencies of class I and II MHC changes was performed with a chi-square test, employing a BMDP statistical software analysis program (Cork, Ireland). *P*-values equal to or lower than 0.01 were regarded to indicate a statistically significant difference.

### Results

### *HPV distribution*

By using general primer (GP5/6)-mediated polymerase chain reaction (PCR) the presence of human papillomavirus (HPV) DNA was assessed in primary tumours of 18 out of the 20 carcinomas analysed. The remaining two samples were PCR negative with both the GP primers and with the human β-globin-specific control primer set, indicating poor target DNA quality. Subsequently, GP-positive samples were subjected to type-specific PCR, resulting in 12 HPV 16-, three HPV 18-, one HPV 31- and one HPV 33-positive cases. One carcinoma did not react with the type-specific primers, and was therefore typed as HPV-X (Table II).

Presence and typing of HPV in the metastasised neoplastic cells in the lymph node was also analysed. No differences were found in HPV types between the primary tumours and lymph nodes.

### *Class I MHC and TAP-1 expression in primary tumours*

After ascertaining general epitope conservation and the epithelial nature of the neoplastic cells with a keratin-specific antibody, immunohistochemical staining for class I MHC expression was analysed with antibodies specific for HLA-A (i.e. HC-A2) and HLA-B/C (i.e. HC10) locus products.

All carcinomas showed similar staining patterns for HLA-A and HLA-B/C expression at the primary tumour site (Table II). An example is given in Figure 1, in which areas of neoplastic cells of carcinoma 70-746 show lack of staining for both HLA-A (Figure 1a) and HLA-B/C (Figure 1b), whereas immune cells in the stroma and within the neoplastic area are labelled for both antigens.

From the 20 primary tumours analysed, four were scored as negative for HLA-A and HLA-B/C, i.e. virtually all neoplastic cells show loss of staining. An additional seven were judged as heterogeneous, i.e. positive tumour areas adjacent to negative ones. The remaining nine carcinomas were scored as positive, i.e. the vast majority of neoplastic cells showing membranous staining.

Expression of one of the subunits of the transporter associated with antigen presentation (TAP-1) was analysed with a polyclonal serum, specific for the TAP-1 protein (Cromme *et al.*, 1993*c*). As shown in Figure 1c, loss of staining for TAP-1 was found in those neoplastic areas of primary tumours that lacked staining for HLA-A and HLA-B/C locus products. This congruency was invariably found in all primary tumours (Table II), which is in accordance with previous findings (Cromme *et al.*, 1993*c*).

### *Class I MHC and TAP-1 expression in lymph node metastases*

In the lymph nodes the same congruency between class I MHC and TAP-1 expression was found (Table II). Metastasised neoplastic cells of carcinoma 76-1849 show loss of HLA-B/C expression (Figure 2b) and staining for TAP-1 (Figure 2c). The same area is negative for HLA-A expression as well (not shown).

However, the frequency of class I MHC and TAP-1 down-regulation was higher in the metastasised neoplastic cells in the lymph nodes than in their primary tumours. An increase in down-regulation of class I MHC expression was found in 12 patients when comparing primary tumours and metastases, as indicated in the MHC-I trend column of Table II. An example is given in Figure 2, in which carcinomas 76-1849 shows positive membranous staining for HLA-B/C at the primary tumour site (Figure 2a), while metastasised cells are clearly negative for HLA-B/C (Figure 2b). In the remaining eight patients the expression patterns in primary tumours and metastases were similar. Two lesions exhibited weak cytoplasmic staining for HLA-A locus products in some (Table II: carcinoma 70-2774) or all (carcinoma 72-831) neoplastic cells. However, since no membranous staining was observed, these were scored as heterogeneous and negative, respectively, for HLA-A cell-surface expression.

For statistical analysis of the difference in MHC and TAP-1 expression between primary tumours and lymph node metastases, heterogeneous and negative expression patterns

**Figure 1** Immunohistochemical staining for class I MHC and TAP-1 expression of primary cervical carcinoma 70-746. **a**, Staining for HLA-A locus products shows positivity in stroma cells and infiltrating immune cells, while neoplastic cells are negative. **b**, Consecutive tissue section of the same primary tumour reveals loss of HLA-B/C locus expression in the same neoplastic area that is negative for HLA-A. Internal staining control cells, i.e. immune cells, are positive. **c**, Identical tumour area is negative for TAP-1 protein. Size bars represent 25 μm.

**Figure 2** Staining for HLA-B/C and TAP-1 in the primary tumour (**a**) and lymph node metastasis (**b** and **c**) of carcinoma 76-1849. **a**, The primary tumour shows positive membranous staining for HLA-B/C locus expression on neoplastic cells. **b**, Metastasised cells in the lymph node from the same patient show a clear lack of HLA-B/C expression, while lymph cells are positive. **c**, The identical neoplastic area is also negative for TAP-1

**Figure 3** Class II MHC expression in primary tumour (**a**) and metastasised cells in the lymph node (**b**) of cervical carcinoma 72-5453, as determined with an HLA-DR-specific antibody. **a**, Neoplastic cells at the primary tumour site show up-regulated HLA-DR expression, with staining localised at the cell membrane. Immune cells in the stroma are positive as well. **b**, Metastasised neoplastic cells of the same patient exhibit no HLA-DR expression, while lymph cells are clearly positive.

were taken together and scored as down-regulated. Such down-regulation was observed in 18 out of 20 metastases, compared with 11 out of 20 primary tumours. This difference is statistically significant by chi-square test (*P*-value 0.01).

*Class II MHC expression in primary tumours*

Class II MHC expression was analysed by immunohistochemistry with an antibody recognising HLA-DR locus products (i.e. LN3). Activated T cells, B cells and macrophages served as internal positive controls, whereas normal squamous epithelium present in the same section was never stained. When a substantial number of neoplastic areas, i.e. 25% or more of the neoplastic cells in a section, stain for HLA-DR, lesions were classified as positive (+). As shown for carcinoma 72-5453 in Figure 3a, neoplastic cells clearly stain positive for HLA-DR at the cell membrane, in addition to positive staining of immune cells in the stroma. In total, nine out of 20 primary tumours were scored as positive for HLA-DR (Table II).

*Class II MHC expression in lymph node metastases*

No HLA-DR expression was observed on neoplastic epithelial cells in the lymph nodes, while lymphocytes in the nodes stained positive. Corresponding tumours at the primary site were scored as positive in nine cases, and an example of this differential staining is shown in Figure 3. Lack of staining for HLA-DR of metastasised neoplastic cells was observed (Figure 3b), while the same patient exhibits up-regulated HLA-DR expression in the primary tumour (Figure 3a). The difference in frequency of HLA-DR expression between primary tumours and metastases is statistically highly significant (*P*-value 0.002).

### Discussion

Certain human papillomavirus (HPV) types are considered to play an important role in cervical carcinogenesis (Zur Hausen, 1989). The major transformation protein E7 from high-risk HPV types (i.e. HPV 16 and 18) interferes with cell cycle control by complexing with the retinoblastoma tumour-suppressor protein (PRb) (Münger *et al.*, 1989), and the E7 open reading frame is consistently transcribed in HPV-containing neoplastic cervical cells (Broker *et al.*, 1989; van den Brule *et al.*, 1991). This viral protein has been shown to be immunogenic *in vitro* (Chen *et al.*, 1991), and immunisation with HPV 16-E7-derived peptide resulted in a protective CTL response in mice (Feltkamp *et al.*, 1993). Therefore the loss of class I MHC surface expression observed in HPV-positive neoplastic cervical cells (Connor & Stern, 1990; Cromme *et al.*, 1993*a*) could be of importance to escape the cellular adaptive immune response.

This study shows that metastasised cervical carcinoma cells displayed a statistically significant increase in frequency of class I MHC down-regulation ($P = 0.01$) as compared with the primary tumour. This observation lends support to the hypothesis that class I MHC-negative tumour cells possess a selective advantage to metastasise and further underlines an important role of the cellular immune response in preventing widespread disease. The latter is also indicated by a 10 times higher risk of developing premalignant cervical lesions (CIN) in women receiving immunosuppressive agents (Sillman & Sedlis, 1987) and the significantly shorter disease-free survival of HIV-seropositive cervical cancer patients (Maiman *et al.*, 1993).

The dominant role for the adaptive immune response in controlling metastasis is further supported by the frequent loss of class I MHC expression *in vivo* during lymph node metastasis in breast, colon, urinary and kidney carcinomas (Cordon-Cardo *et al.*, 1991) and in melanomas (Lopez-Nevot *et al.*, 1986; Ruiter *et al.*, 1986). On the other hand, several reports have shown that an increase in expression of class I MHC results in a higher number of metastases in mice (De Baetselier *et al.*, 1980; Katzav *et al.*, 1984; Algarra *et al.*, 1991). This could be due to a decreased natural killer (NK)-cell susceptibility of class I MHC-positive cells, according to the hypothesis that NK cells recognise 'missing self' (Kärre *et al.*, 1986). However, restoration of class I MHC expression by γ-interferon treatment of melanoma cells reduces their metastatic potential (Zöller *et al.*, 1988), indicating that the increased efficacy of T-cell response can overcome the loss of a non-adaptive immune defence *in vitro*.

Recently we have shown that the down-regulation of class I MHC expression is strongly associated with loss of the transporter associated with antigen presentation (TAP) in primary cervical carcinomas (Cromme *et al.*, 1993*c*). This fits into the model of post-transcriptional regulation of class I MHC expression (Cromme *et al.*, 1993*b*), in which lack of class I MHC stabilisation in the ER occurs when peptide levels are reduced owing to loss of TAP. The observation in this study that the increased frequency of class I MHC down-regulation in lymph node metastases is consistently accompanied by loss of TAP-1 protein confirms and extends the relationship between the expression of HLA and TAP-1 and indicates a role during tumorigenesis *in vivo*. The regulation of TAP-1 documented in this and other studies (Cromme *et al.*, 1993*c*; Restifo *et al.*, 1993) may not therefore be epiphenomenon but a target for selection during progression of malignant disease. Further investigation into the mechanisms of TAP-1 down-regulation could provide the tool to restore antigen presentation and consequently render neoplastic cells sensitive to $CD8^+$ CTL-mediated immune therapy.

Two patients in this study showed no down-regulation of class I MHC expression in either the primary tumour site or the lymph node, but were HLA-DR positive in the primary tumour. Presumably other mechanisms of immune escape can operate in these cases. Under some circumstances up-regulated class II MHC expression on keratinocytes can result in T-cell anergy or tolerance, presumably due to an incorrect antigen presentation (Gaspari *et al.*, 1988; Bal *et al.*, 1990). Therefore *de novo* HLA-DR expression on neoplastic epithelial cells as observed in this and other studies (Glew *et al.*, 1992; Cromme *et al.*, 1993*a*) may have induced a state of T-cell anergy.

Alternatively, class II MHC molecules may serve as restriction elements for tumour-specific $CD4^+$ CTLs. In line with this is the finding that peripheral $CD4^+$ T cells in HPV-seropositive donors can lyse autologous B cells after pulsing with HPV 16-E7-encoded peptides, presumably in a class II MHC-restricted fashion (Altmann *et al.*, 1992). Provided that endogenous HPV proteins can enter the class II secretory pathway *in vivo*, in analogy with cytosolic vaccinia proteins (Jaraquemada *et al.*, 1990), the up-regulated HLA-DR expression could result in specific lysis of cervical tumour cells by $CD4^+$ T cells. This would explain selection for HLA-DR-negative tumour cells during metastasis.

However, it cannot be excluded that the difference in frequency of HLA-DR expression between primary tumours and lymph node metastases is related to differences in local cytokine production (i.e. γ-interferon, tumour necrosis factor) and therefore needs to be considered as a bystander effect.

Further research into the nature of infiltrating immune cells and their state of activation is required to establish which immune cell population has been primed. This could further elucidate which target cell recognition structures are negatively selected for during cervical carcinogenesis.

Authors would like to thank J.J. Neefjes for supplying antibodies HC10 and HC-A2, H.L. Ploegh for supplying polyclonal TAP-1 serum and M.J. Stukart for critical reading of the manuscript. This work was supported by grants from the Prevention Fund (28-1502.2) and the Dutch Cancer Society (IKA 91-10), The Netherlands. P.L. Stern was supported by the Cancer Research Campaign of Great Britain.

### References

ALGARRA, I., GAFORIO, J.J., GARRIDO, A., MIALDEA, M.J., PEREZ, M. & GARRIDO, F. (1991). Heterogeneity of MHC class-I antigens in clones of methylcholanthrene-induced tumours. Implications for local growth and metastasis. *Int. J. Cancer*, **6** (Suppl.), 73–81.

ALTMANN, A., JOCHMUS-KUDIELKA, I., FRANK, R., GAUSEPOHL, H., MOEBIUS, U., GISSMANN, L. & MEUER, S.C. (1992). Definition of immunogenic determinants of the human papillomavirus type 16 nucleoprotein E7. *Eur. J. Cancer*, **28**, 326–333.

BAL, V., MCINDOE, A., DENTON, G., HUDSON, D., LOMBARDI, G., LAMB, J. & LECHLER, R. (1990). Antigen presentation by keratinocytes induces tolerance in human T-cells. *Eur. J. Immunol.*, **20**, 1893–1897.

BROKER, T.R., CHOW, L.T., CHIN, M.T., RHODES, C.R., WOLINSKY, S.M., WHITBECK, A. & STOLER, M.H. (1989). A molecular portrait of human papillomavirus carcinogenesis. *Cancer Cells*, **7**, 197.

CHEN, L., THOMA, E.K., HU, S.L., HELLSTROM, I. & HELLSTROM, K.E. (1991). Human papillomavirus type 16 nucleoprotein E7 is a tumour rejection antigen. *Proc. Natl Acad. Sci. USA*, **88**, 110–114.

CONNOR, M.E. & STERN, P.L. (1990). Loss of MHC class-I expression in cervical carcinomas. *Int. J. Cancer*, **46**, 1029–1034.

CONCHA, A., CABRERA, T., RUIZ-CABELLO, F. & GARRIDO, F. (1991). Can the HLA phenotype be used as a prognostic factor in breast carcinomas? *Int. J. Cancer*, **6** (Suppl.), 146–154.

CORDON-CARDO, C., FUKS, Z., DROBNJAK, M., MORENO, C., EISENBACH, L. & FELDMAN, M. (1991). Expression of HLA-A,B,C antigens on primary and metastatic tumour cell populations of human carcinomas. *Cancer Res.*, **51**, 6372–6380.

CROMME, F.V., MEIJER, C.J.L.M., SNIJDERS, P.J.F., UYTERLINDE, A., KENEMANS, P., HELMERHORST, TH., STERN, P.L., VAN DEN BRULE, A.J.C. & WALBOOMERS, J.M.M. (1993*a*). Analysis of MHC class I and II expression in relation to presence of HPV genotypes in premalignant and malignant cervical lesions. *Br. J. Cancer*, **67**, 1372–1380.

CROMME, F.V., SNIJDERS, P.J.F., VAN DEN BRULE, A.J.C., KENEMANS, P., MEIJER, C.J.L.M. & WALBOOMERS, J.M.M. (1993*b*). MHC class I expression in HPV 16 positive cervical carcinomas is post-transcriptionally controlled and independent from c-myc overexpression. *Oncogene*, **8**, 2969–2975.

CROMME, F.V., AIREY, J., HEEMELS, M.-T., PLOEGH, H.L., KEATING, P.J., STERN, P.L., MEIJER, C.J.L.M. & WALBOOMERS, J.M.M. (1993*c*). Loss of transporter protein, encoded by the TAP-1 gene, is highly correlated with loss of HLA expression in cervical carcinomas. *J. Exp. Med.* (in press).

DE BAETSELIER, P.D., KATZAV, S., GORELIK, M., FELDMAN, M. & SEGAL, S. (1980). Differential expression of H2 gene products in tumour cells is associated with their metastatic properties. *Nature*, **288**, 179–181.

ESTEBAN, F., CONCHA, A., HUELIN, C., PEREZ-AYALA, M., PEDRINACI, S., RUIZ-CABELLO, F. & GARRIDO, F. (1989). Histocompatibility antigens in primary and metastatic squamous cell carcinomas of the larynx. *Int. J. Cancer*, **43**, 436–442.

ESTEBAN, F., RUIZ-CABELLO, F., CONCHAS, A., PEREZ-AYALA, M., SANCHEZ-ROSAS, J.A. & GARRIDO, F. (1990). HLA-DR expression is associated with excellent prognosis in squamous cell carcinomas of the larynx. *Clin. Exp. Metastases*, **8**, 319–328.

FALK, K., RÖTSCHKE, O. & RAMMENSEE, H.-G. (1990). Cellular peptide composition is governed by major histocompatibility complex class I molecules. *Nature*, **348**, 248–251.

FELTKAMP, M.C.W., SMITS, H.L., VIERBOOM, M.P.M., MINNAAR, R.P., DE JONGH, B.M., DRIJFHOUT, J.W., TER SCHEGGET, J., MELIEF, C.J.M. & KAST, W.M. (1993). Peptide vaccination with a cytotoxic T cell epitope derived from the human papillomavirus type 16 oncogene E7 confers protection against HPV16 induced tumours. *J. Cell. Biochem.*, **17D** (Suppl.), 93.

GASPARI, A., JENKINS, M.K. & KATZ, S.I. (1988). Class II MHC-bearing keratinocytes induce antigen-specific unresponsiveness in hapten-specific TH1 clones. *J. Immunol.*, **141**, 2216–2220.

GLEW, S.S., DUGGAN-KEEN, M., CABRERA, T. & STERN, P.L. (1992). HLA class II antigen expression in human papillomavirus-associated cervical cancer. *Cancer Res.*, **52**, 4009–4016.

GLEW, S.S., CONNOR, M.E., SNIJDERS, P.J.F., STANBRIDGE, C., BUCKLEY, C.H., WALBOOMERS, J.M.M., MEIJER, C.J.L.M. & STERN, P.L. (1993). HLA expression in pre-invasive cervical neoplasia in relationship to human papillomavirus infection. *Eur. J. Cancer.*, **29A**, 1963–1970.

GOLDBERG, A.L. & ROCK, K.L. (1992). Proteolysis, proteasomes and antigen presentation. *Nature*, **357**, 375–378.

JARAQUEMADA, D., MARTI, M. & LONG, E.O. (1990). An endogenous processing pathway in vaccinia virus-infected cells for presentation of cytoplasmic antigens to class II-restricted T-cells. *J. Exp. Med.*, **172**, 947–954.

KÄRRE, K., LJUNGGREN, H.G., PIONTEK, G. & KIESSLING, R. (1986). Selective rejection of H2-deficient lymphoma variants suggests alternative immune defense strategy. *Nature*, **319**, 675–678.

KATZAV, S., SEGAL, S. & FELDMAN, M. (1984). Immunoselection in vivo of H2-D phenotypic variants from a metastatic clone of sarcoma cells result in cell lines of altered metastatic competence. *Int. J. Cancer*, **33**, 407–415.

LOPEZ-NEVOT, M.A., GARCIA, E., PAREJA, E., BONAL, F.J., MARTIN, J., RUIZ-CABELLO, F. & GARRIDO, F. (1986). Differential expression of HLA class I and II antigens in primary and metastatic melanomas. *J. Immunogenet.*, **13**, 219–227.

MAIMAN, M., FRUCHTER, R.G., GUY, L., CUTHILL, S., LEVINE, P. & SERUR, E. (1993). Human immunodeficiency virus infection and invasive cervical carcinoma. *Cancer*, **71**, 402–406.

MÜNGER, K., WERNESS, B.A., DYSON, N., PHELPS, W.C. & HOWLEY, P.M. (1989). Complex formation of human papillomavirus E7 proteins with the retinoblastoma tumour suppressor gene product. *EMBO J.*, **8**, 4099–4105.

NEEFJES, J.J., MOMBURG, F. & HÄMMERLING, G.J. (1993). Selective and ATP-dependent translocation of peptides by the MHC-encoded transporter. *Science*, **261**, 769–771.

PATERSON, A.C., SCOIT, R., KEW, M.C., CALLEA, F., DUSKEIKO, G.M. & DESMET, V.G. (1988). HLA expression in human hepatocellular carcinoma. *Br. J. Cancer*, **57**, 369–373.

RESTIFO, N.P., ESQUIVEL, F., KAWAKAMI, Y., YEWDELL, J.W., MULE, J.J., ROSENBERG, S.A. & BENNINK, J.R. (1993). Identification of human cancers deficient in antigen processing. *J. Exp. Med.*, **177**, 265–272.

RUITER, D.J., BROCKER, E.B. & FERRONE, S. (1986). Expression and susceptibility to modulation by interferons of HLA class I and II antigens in melanoma cells. Immunohistochemical analysis and clinical relevance. *J. Immunogenet.*, **13**, 229–234.

RUIZ-CABELLO, F., KLEIN, E. & GARRIDO, F. (1991). MHC antigens on human tumours. *Immunol. Lett.*, **29**, 181–190.

SAKAI, K., TAKIGUCHI, M., MORI, S., KOBORI, O., MORIOKA, Y., INOKO, H., SERIGUCHI, M. & KANO, K. (1987). Expression and function of class II MHC antigens on gastric carcinoma cells and gastric epithelia: differential expression of DR, DP and DQ antigens. *J. Natl Cancer Inst.*, **79**, 923–932.

SHEPHERD, J.C., SCHUMACHER, T.N.M., ASHTON-RICKARDT, P.G., IMAEDA, S., PLOEGH, H.L., JANEWAY, C.A. & TONEGAWA, S. (1993). TAP1-dependent peptide translocation in vitro is ATP dependent and peptide selective. *Cell*, **74**, 577–584.

SILLMAN, F.H. & SEDLIS, A. (1987). Anogenital papillomavirus infection and neoplasia in immunodeficient women. In *Human Papillomavirus*, Reed, R. (ed.) pp. 537–558. W.B. Saunders: Philadelphia.

SNIJDERS, P.J.F., VAN DEN BRULE, A.J.C., SCHRIJNEMAKERS, H.F.J., SNOW, G., MEIJER, C.J.L.M. & WALBOOMERS, J.M.M. (1990). The use of general primers in the polymerase chain reaction permits the detection of a broad spectrum of human papillomavirus genotypes. *J. Gen. Virol.*, **71**, 173–181.

SPIES, T., BRESNAHAN, M., BAHRAM, S., ARNOLD, D., BLANCK, G., MELLINS, E., PIOUS, D. & DEMARS, R. (1990). A gene in the human major histocompatibility complex class II region controlling the class I antigen presentation pathway. *Nature*, **348**, 744–747.

STAM, N.J., SPITS, H. & PLOEGH, H.L. (1986). Monoclonal antibodies raised against denatured HLA-B locus heavy chains permit biochemical characterisation of certain HLA-C locus products. *J. Immunol.*, **137**, 2299–2306.

TOWNSEND, A.R.M., GOTCHI, F.M. & DAVEY, J. (1985). Cytotoxic T-cells recognize fragments of the influenza protein. *Cell*, **42**, 457–467.

TROWSDALE, J., HANSON, I., MOCKRIDGE, I., BECK, S., TOWNSEND, A. & KELLY, A. (1990). Sequences encoded in the class II region of MHC related to the 'ABC' superfamily of transporters. *Nature*, **348**, 741–743.

VAN DEN BRULE, A.J.C., MEIJER, C.J.L.M., BAKELS, V., KENEMANS, P. & WALBOOMERS, J.M.M. (1990). Rapid human papillomavirus detection in cervical scrapes by combined general primer-mediated and type-specific polymerase chain reaction. *J. Clin. Microbiol.*, **28**, 2739–2743.

VAN DEN BRULE, A.J.C., CROMME, F.V., SNIJDERS, P.J.F., SMIT, L., OUDEJANS, C.B.M., BAAK, J.P.A., MEIJER, C.J.L.M. & WALBOOMERS, J.M.M. (1991). Non radioactive RNA in situ hybridisation detection of HPV-16 E7 transcripts in squamous cell carcinomas of the uterine cervix using confocal laserscan microscopy. *Am. J. Pathol.*, **139**(5), 1037–1045.

VAN DEN INGH, H.F., RUITER, F.K., GRIFFIOEN, G., VAN MIUJEN, G.N.P. & FERRONE, S. (1987). HLA antigens in colorectal tumours: low expression of HLA class I antigens in mucinous colorectal carcinomas. *Br. J. Cancer*, **55**, 125–130.

WALBOOMERS, J.M.M., MELKERT, P.W.J., VAN DEN BRULE, A.J.C., SNIJDERS, P.J.F. & MEIJER, C.J.L.M. (1992). The polymerase chain reaction for screening in diagnostic cytopathology of the cervix. In *Diagnostic Molecular Pathology*, Vol. 2, Herrington, C.S. & McGee, O.D. (eds) pp. 153–172. IRL Press: Oxford.

ZÖLLER, M., STRUBEL, A., HÄMMERLING, G., ANDRIGHETTO, G., RAZ, A. & BEN-ZE'EV, A. (1988). Interferon-gamma treatment of B16 melanoma cells: opposing effects for non-adaptive and adaptive immune defense and its reflection by metastatic spread. *Int. J. Cancer*, **41**, 256–266.

ZUR HAUSEN, H. (1989). Papillomaviruses in anogenital cancer as a model to understand the role of viruses in human cancer. *Cancer Res.*, **49**, 4677–4681.


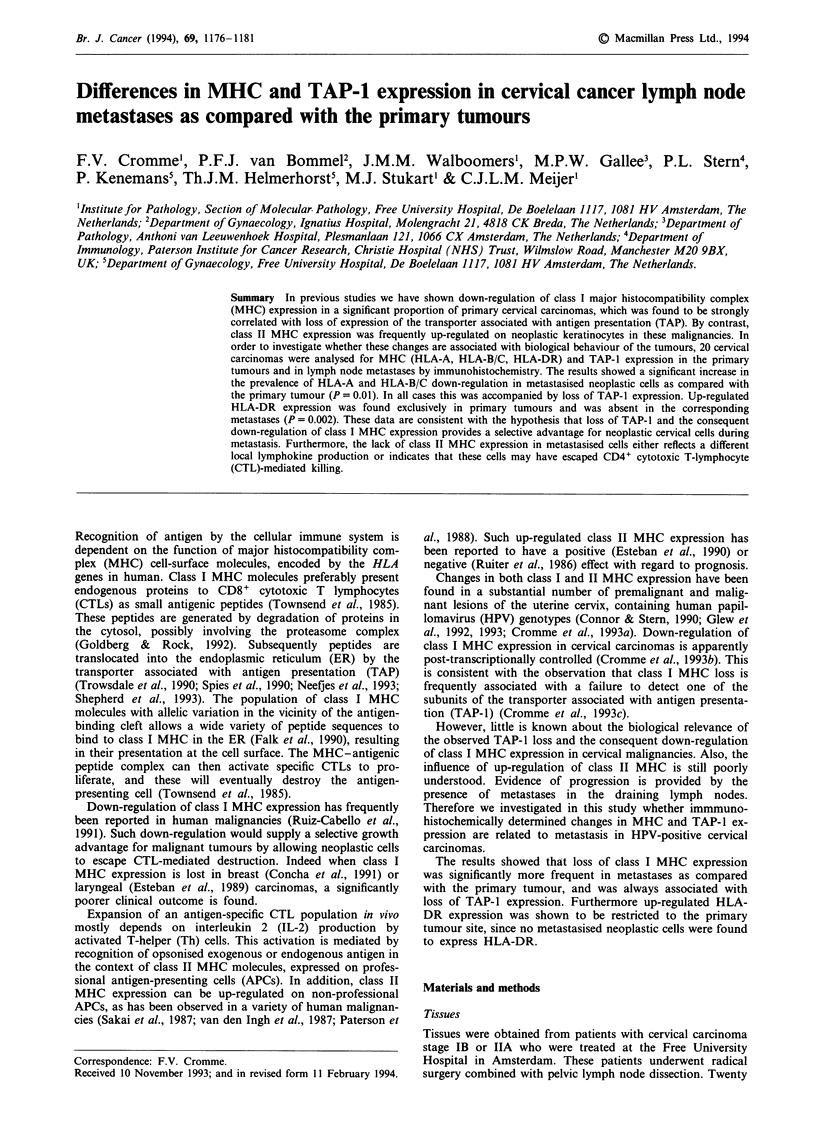

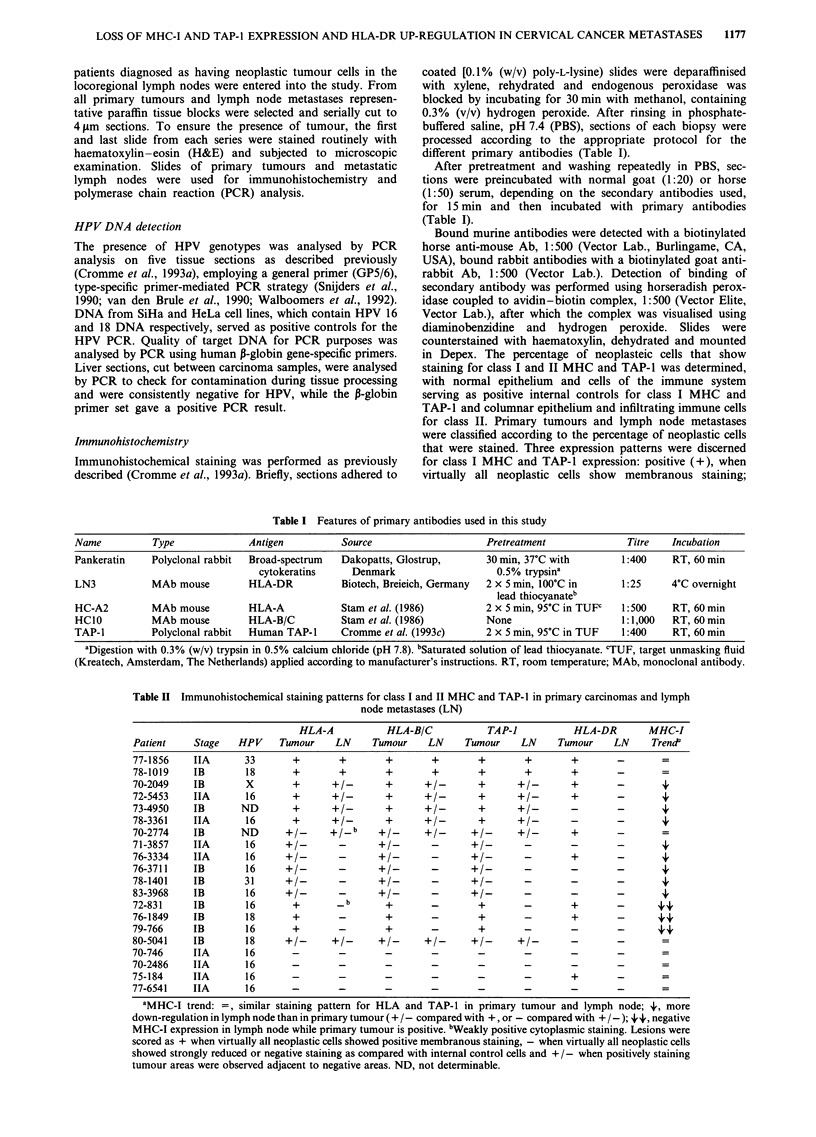

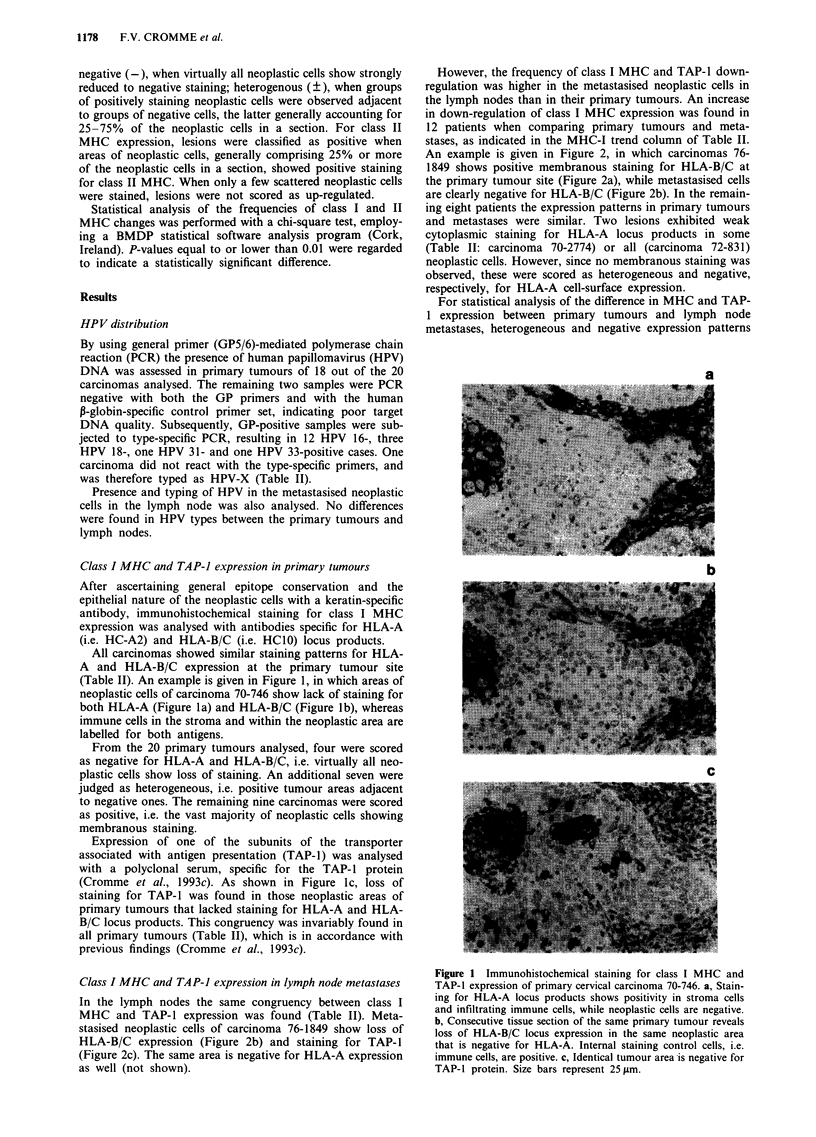

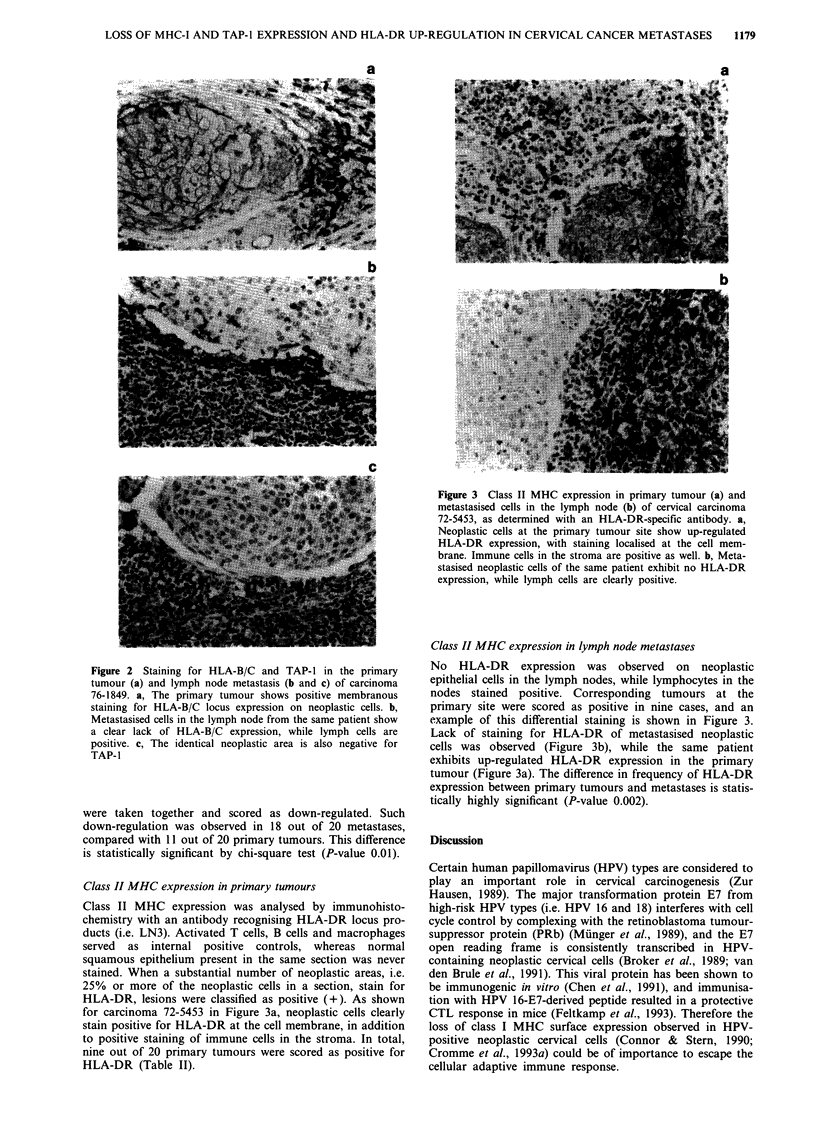

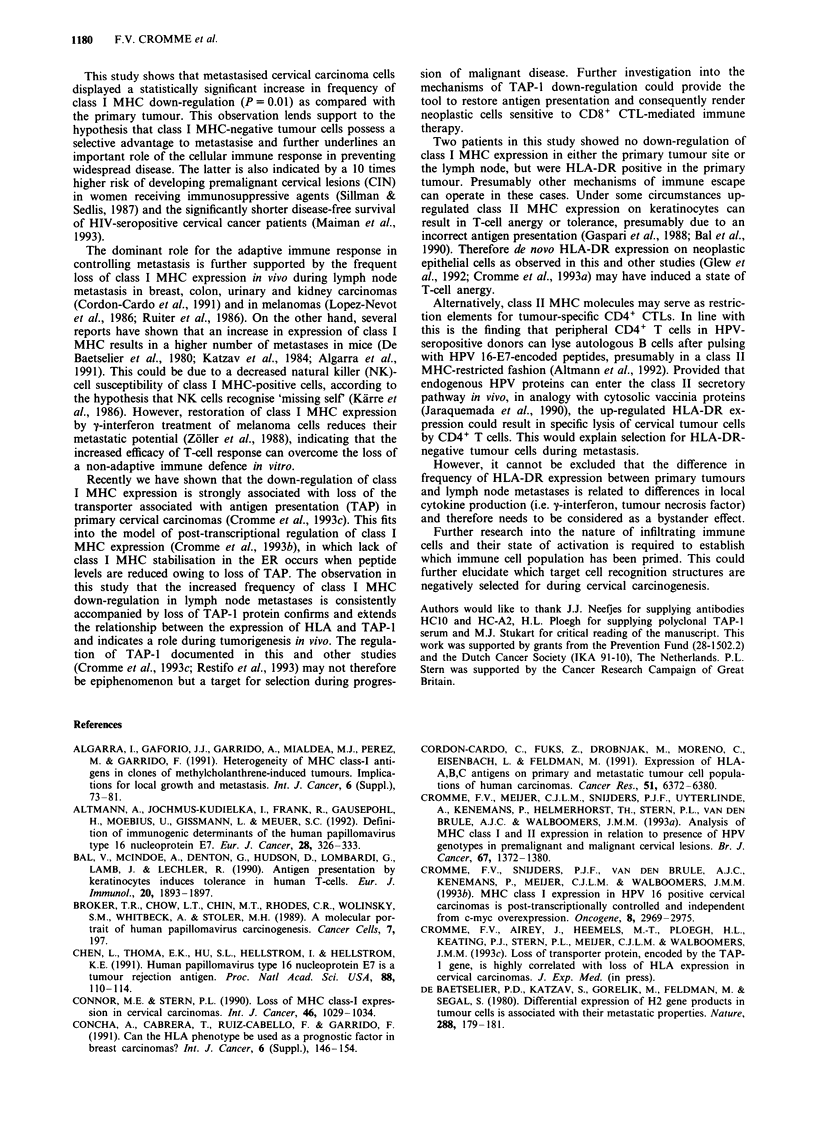

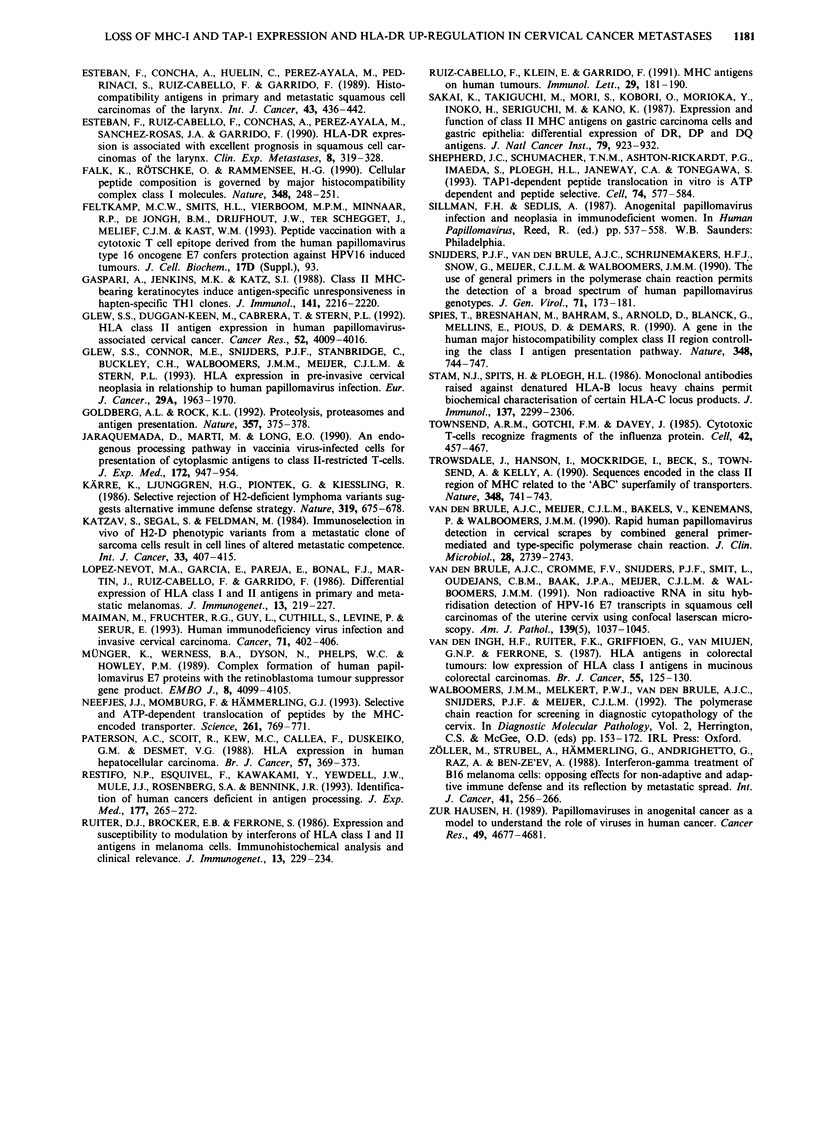


## References

[OCR_00556] Algarra I., Gaforio J. J., Garrido A., Mialdea M. J., Pérez M., Garrido F. (1991). Heterogeneity of MHC-class-I antigens in clones of methylcholanthrene-induced tumors. Implications for local growth and metastasis.. Int J Cancer Suppl.

[OCR_00563] Altmann A., Jochmus-Kudielka I., Frank R., Gausepohl H., Moebius U., Gissmann L., Meuer S. C. (1992). Definition of immunogenic determinants of the human papillomavirus type 16 nucleoprotein E7.. Eur J Cancer.

[OCR_00569] Bal V., McIndoe A., Denton G., Hudson D., Lombardi G., Lamb J., Lechler R. (1990). Antigen presentation by keratinocytes induces tolerance in human T cells.. Eur J Immunol.

[OCR_00581] Chen L. P., Thomas E. K., Hu S. L., Hellström I., Hellström K. E. (1991). Human papillomavirus type 16 nucleoprotein E7 is a tumor rejection antigen.. Proc Natl Acad Sci U S A.

[OCR_00591] Concha A., Cabrera T., Ruiz-Cabello F., Garrido F. (1991). Can the HLA phenotype be used as a prognostic factor in breast carcinomas?. Int J Cancer Suppl.

[OCR_00587] Connor M. E., Stern P. L. (1990). Loss of MHC class-I expression in cervical carcinomas.. Int J Cancer.

[OCR_00596] Cordon-Cardo C., Fuks Z., Drobnjak M., Moreno C., Eisenbach L., Feldman M. (1991). Expression of HLA-A,B,C antigens on primary and metastatic tumor cell populations of human carcinomas.. Cancer Res.

[OCR_00605] Cromme F. V., Meijer C. J., Snijders P. J., Uyterlinde A., Kenemans P., Helmerhorst T., Stern P. L., van den Brule A. J., Walboomers J. M. (1993). Analysis of MHC class I and II expression in relation to presence of HPV genotypes in premalignant and malignant cervical lesions.. Br J Cancer.

[OCR_00610] Cromme F. V., Snijders P. J., van den Brule A. J., Kenemans P., Meijer C. J., Walboomers J. M. (1993). MHC class I expression in HPV 16 positive cervical carcinomas is post-transcriptionally controlled and independent from c-myc overexpression.. Oncogene.

[OCR_00624] De Baetselier P., Katzav S., Gorelik E., Feldman M., Segal S. (1980). Differential expression of H-2 gene products in tumour cells in associated with their metastatogenic properties.. Nature.

[OCR_00634] Esteban F., Concha A., Huelin C., Pérez-Ayala M., Pedrinaci S., Ruiz-Cabello F., Garrido F. (1989). Histocompatibility antigens in primary and metastatic squamous cell carcinoma of the larynx.. Int J Cancer.

[OCR_00638] Esteban F., Ruiz-Cabello F., Concha A., Pérez-Ayala M., Sánchez-Rozas J. A., Garrido F. (1990). HLA-DR expression is associated with excellent prognosis in squamous cell carcinoma of the larynx.. Clin Exp Metastasis.

[OCR_00644] Falk K., Rötzschke O., Rammensee H. G. (1990). Cellular peptide composition governed by major histocompatibility complex class I molecules.. Nature.

[OCR_00657] Gaspari A. A., Jenkins M. K., Katz S. I. (1988). Class II MHC-bearing keratinocytes induce antigen-specific unresponsiveness in hapten-specific Th1 clones.. J Immunol.

[OCR_00667] Glew S. S., Connor M. E., Snijders P. J., Stanbridge C. M., Buckley C. H., Walboomers J. M., Meijer C. J., Stern P. L. (1993). HLA expression in pre-invasive cervical neoplasia in relation to human papilloma virus infection.. Eur J Cancer.

[OCR_00662] Glew S. S., Duggan-Keen M., Cabrera T., Stern P. L. (1992). HLA class II antigen expression in human papillomavirus-associated cervical cancer.. Cancer Res.

[OCR_00674] Goldberg A. L., Rock K. L. (1992). Proteolysis, proteasomes and antigen presentation.. Nature.

[OCR_00678] Jaraquemada D., Marti M., Long E. O. (1990). An endogenous processing pathway in vaccinia virus-infected cells for presentation of cytoplasmic antigens to class II-restricted T cells.. J Exp Med.

[OCR_00688] Katzav S., Segal S., Feldman M. (1984). Immuno-selection in vivo of H-2D phenotypic variants from a metastatic clone of sarcoma cells results in cell lines of altered metastatic competence.. Int J Cancer.

[OCR_00684] Kärre K., Ljunggren H. G., Piontek G., Kiessling R. (1986). Selective rejection of H-2-deficient lymphoma variants suggests alternative immune defence strategy.. Nature.

[OCR_00696] Lopez Nevot M. A., Garcia E., Pareja E., Bonal F. J., Martin J., Ruiz-Cabello F., Serrano S., Garrido F. (1986). Differential expression of HLA class I and II antigens in primary and metastatic melanomas.. J Immunogenet.

[OCR_00700] Maiman M., Fruchter R. G., Guy L., Cuthill S., Levine P., Serur E. (1993). Human immunodeficiency virus infection and invasive cervical carcinoma.. Cancer.

[OCR_00705] Münger K., Werness B. A., Dyson N., Phelps W. C., Harlow E., Howley P. M. (1989). Complex formation of human papillomavirus E7 proteins with the retinoblastoma tumor suppressor gene product.. EMBO J.

[OCR_00711] Neefjes J. J., Momburg F., Hämmerling G. J. (1993). Selective and ATP-dependent translocation of peptides by the MHC-encoded transporter.. Science.

[OCR_00716] Paterson A. C., Sciot R., Kew M. C., Callea F., Dusheiko G. M., Desmet V. J. (1988). HLA expression in human hepatocellular carcinoma.. Br J Cancer.

[OCR_00721] Restifo N. P., Esquivel F., Kawakami Y., Yewdell J. W., Mulé J. J., Rosenberg S. A., Bennink J. R. (1993). Identification of human cancers deficient in antigen processing.. J Exp Med.

[OCR_00727] Ruiter D. J., Bröcker E. B., Ferrone S. (1986). Expression and susceptibility to modulation by interferons of HLA class I and II antigens on melanoma cells. Immunohistochemical analysis and clinical relevance.. J Immunogenet.

[OCR_00733] Ruiz-Cabello F., Klein E., Garrido F. (1991). MHC antigens on human tumors.. Immunol Lett.

[OCR_00737] Sakai K., Takiguchi M., Mori S., Kobori O., Morioka Y., Inoko H., Sekiguchi M., Kano K. (1987). Expression and function of class II antigens on gastric carcinoma cells and gastric epithelia: differential expression of DR, DQ, and DP antigens.. J Natl Cancer Inst.

[OCR_00744] Shepherd J. C., Schumacher T. N., Ashton-Rickardt P. G., Imaeda S., Ploegh H. L., Janeway C. A., Tonegawa S. (1993). TAP1-dependent peptide translocation in vitro is ATP dependent and peptide selective.. Cell.

[OCR_00750] Sillman F. H., Sedlis A. (1987). Anogenital papillomavirus infection and neoplasia in immunodeficient women.. Obstet Gynecol Clin North Am.

[OCR_00758] Snijders P. J., van den Brule A. J., Schrijnemakers H. F., Snow G., Meijer C. J., Walboomers J. M. (1990). The use of general primers in the polymerase chain reaction permits the detection of a broad spectrum of human papillomavirus genotypes.. J Gen Virol.

[OCR_00763] Spies T., Bresnahan M., Bahram S., Arnold D., Blanck G., Mellins E., Pious D., DeMars R. (1990). A gene in the human major histocompatibility complex class II region controlling the class I antigen presentation pathway.. Nature.

[OCR_00770] Stam N. J., Spits H., Ploegh H. L. (1986). Monoclonal antibodies raised against denatured HLA-B locus heavy chains permit biochemical characterization of certain HLA-C locus products.. J Immunol.

[OCR_00776] Townsend A. R., Gotch F. M., Davey J. (1985). Cytotoxic T cells recognize fragments of the influenza nucleoprotein.. Cell.

[OCR_00783] Trowsdale J., Hanson I., Mockridge I., Beck S., Townsend A., Kelly A. (1990). Sequences encoded in the class II region of the MHC related to the 'ABC' superfamily of transporters.. Nature.

[OCR_00815] Zöller M., Strubel A., Hämmerling G., Andrighetto G., Raz A., Ben-Ze'ev A. (1988). Interferon-gamma treatment of B16 melanoma cells: opposing effects for non-adaptive and adaptive immune defense and its reflection by metastatic spread.. Int J Cancer.

[OCR_00797] van den Brule A. J., Cromme F. V., Snijders P. J., Smit L., Oudejans C. B., Baak J. P., Meijer C. J., Walboomers J. M. (1991). Nonradioactive RNA in situ hybridization detection of human papillomavirus 16-E7 transcripts in squamous cell carcinomas of the uterine cervix using confocal laser scan microscopy.. Am J Pathol.

[OCR_00787] van den Brule A. J., Meijer C. J., Bakels V., Kenemans P., Walboomers J. M. (1990). Rapid detection of human papillomavirus in cervical scrapes by combined general primer-mediated and type-specific polymerase chain reaction.. J Clin Microbiol.

[OCR_00802] van den Ingh H. F., Ruiter D. J., Griffioen G., van Muijen G. N., Ferrone S. (1987). HLA antigens in colorectal tumours--low expression of HLA class I antigens in mucinous colorectal carcinomas.. Br J Cancer.

[OCR_00822] zur Hausen H. (1989). Papillomaviruses in anogenital cancer as a model to understand the role of viruses in human cancers.. Cancer Res.

